# Automated fruit maturity grading using deep learning with feature fusion

**DOI:** 10.1038/s41598-026-54872-8

**Published:** 2026-06-05

**Authors:** S. Kripa, V. Jeyalakshmi

**Affiliations:** Department of Electronics and Communication Engineering, College of Engineering Guindy, Anna University, Chennai, Tamil Nadu 600025 India

**Keywords:** Maturity grading, Internal attributes, External attributes, k-means clustering, Convolution neural network, Feature fusion, Computational biology and bioinformatics, Engineering, Mathematics and computing, Plant sciences

## Abstract

Fruits are valued for their nutritional benefits, providing essential carbohydrates, vitamins, and dietary fibre. However, assessing fruit ripeness remains challenging, as it is governed by complex physiological processes and environmental factors that are not always reflected in external appearance. This difficulty is exemplified by Nam Dok Mai Si Tong (NDMST) mangoes, which retain a largely uniform yellow skin during ripening, making visual maturity grading unreliable. To address this limitation, we propose AFMG-DLFF (Automated Fruit Maturity Grading using Deep Learning with Feature Fusion), a multimodal deep learning framework that integrates external RGB image features with internal biochemical attributes. Visual features are extracted using DenseNet201, Inception–ResNetV2, and EfficientNetV2, while intrinsic traits such as total soluble solids, titratable acidity, and BrimA are encoded via a dedicated neural network. These complementary feature spaces are fused and optimised using Glowworm Swarm Optimization (GSO) for hyperparameter tuning. The model is trained with an 80:20 train–test split and early-stopping-based validation, achieving a classification accuracy of 97.86% for NDMST mango maturity stages. The results demonstrate that AFMG-DLFF achieves strong performance relative to the evaluated deep-learning baselines and remains competitive with selected literature-reported fruit ripeness classification methods, while relying only on accessible RGB imaging and standard biochemical measurements. This highlights its potential as a practical, non-destructive, and cost-effective solution for automated fruit maturity grading in real-world supply chains.

## Introduction

The agricultural sector plays a pivotal role in global food security, and recent advances in smart farming - spanning IoT devices, sensing technologies, data analytics, artificial intelligence, and automation have transformed how crops are monitored, harvested, and distributed^1^. Among the various challenges in this domain, the accurate assessment of fruit maturity remains crucial for ensuring optimal quality, reducing postharvest losses, and improving supply-chain efficiency. Traditionally, fruit maturity is evaluated through external visual cues such as skin colour, size, shape, and texture. However, these cues may be unreliable for certain cultivars or unfamiliar fruit varieties, making manual grading subjective, labour-intensive, and inconsistent.

Mango is one of the most commercially significant fruits worldwide, yet several varieties present difficulties in visual maturity determination. A notable example is NDMST, a premium Thai cultivar that retains a uniform yellow peel throughout ripening due to bagging practices applied during development^2,3^. As a result, NDMST mangoes lack the green-to-yellow transformation typical of many cultivars, rendering colour-based assessment ineffective. Furthermore, fruits with similar external appearances may differ significantly in internal composition due to environmental factors, regional variation, and microclimatic influences. These limitations highlight the need for methods that incorporate internal indicators of ripeness—such as aroma, moisture content, total soluble solids (TSS), and titratable acidity (TA) which provide more reliable insights into physiological maturity^4^.

Recent advancements in non-destructive technologies, including NIR spectroscopy^5^, magnetic resonance imaging (MRI), hyperspectral imaging (HSI), and acoustic sensing, have enabled improved evaluation of fruit internal quality. However, these modalities often require expensive instrumentation and extensive computational resources, making them impractical for routine grading in most agricultural settings. This motivates the development of hybrid approaches that combine low-cost imaging with key biochemical indicators to deliver robust and accessible maturity assessment.

In parallel, the rise of machine learning and deep learning has considerably enhanced the ability to extract discriminative features from visual and biological data^6^. Convolutional neural networks (CNNs) and related architectures have demonstrated strong performance across a broad range of agricultural tasks, providing objective, scalable, and real-time capabilities. Motivated by these advances, this work introduces AFMG-DLFF, an automated multimodal fruit maturity grading framework designed specifically for NDMST mangoes. Unlike traditional systems that rely solely on external attributes or require specialised imaging equipment, AFMG-DLFF integrates RGB visual features with intrinsic biochemical traits to deliver an end-to-end classification of unripe, semi-ripe, and ripe stages. Through multimodel feature fusion and the combination of extrinsic and intrinsic modalities, the proposed system provides a comprehensive, and practical maturity assessment solution.

### Highlights

The key contributions of this work are as follows:

1. *NDMST maturity grading problem:* We address the unique challenge posed by NDMST mangoes, whose visually indistinct ripening stages render conventional colour-based grading ineffective. By jointly analysing external and internal biological parameters, the proposed framework significantly improves the precision of maturity-stage identification.
2. *Multimodel visual feature fusion:* The framework introduces a novel fusion strategy that leverages three pretrained CNNs: DenseNet201, Inception–ResNetV2, and EfficientNetV2 to capture both fine-grained and holistic visual characteristics. This multimodel fusion enhances discriminative capacity for external trait representation.
3. *Integrative extrinsic–intrinsic fusion:* Beyond visual cues, AFMG-DLFF incorporates a biochemical feature vector derived from TSS, TA, and BrimA measurements. The fusion of external appearance with internal physiological signatures yields a richer and more reliable representation for maturity classification.
4. *Effective grading with limited datasets:* The method demonstrates strong performance despite limited training data. Through CGAN-based image augmentation, SMOTE-based biochemical balancing, and GSO-guided optimisation, the proposed model achieves robust classification accuracy and demonstrates resilience in data-constrained scenarios.

The remainder of this paper is organised as follows: “Related work” section reviews related work; “Methodology” details the proposed AFMG-DLFF framework; “Empirical findings and discussions” presents experimental results and discussion; and “Conclusion” concludes the study.

## Related work

Fruit grading is traditionally performed using two complementary approaches: (i) analysing external visual attributes and (ii) evaluating internal biological metrics that reflect physiological maturity. This reviews prior work in both dimensions and outlines the motivation for a multimodal fusion strategy.

### Grading with external analysis

Visual appearance is one of the most widely used indicators of fruit quality, with attributes such as skin colour, surface bruises, and texture playing a central role in consumer perception and commercial grading^7^. Modern fruit grading systems increasingly rely on image processing, machine learning, and deep learning techniques to improve objectivity, consistency, and operational efficiency^8,9^. These methods also reduce labour requirements and processing time, positioning them as cost-effective solutions for large-scale postharvest applications.

Extensive research has explored computer vision methods for mango quality assessment. Doan et al.^10^ applied Otsu thresholding, contour extraction, and Canny edge detection to identify surface imperfections. Yeh et al.^11^ proposed a four-module grading system incorporating Mask R-CNN for segmentation, achieving high accuracy and enabling real-time sorting. Dairath et al.^12^ combined CNNs with fuzzy logic, KNN, and SVM to detect fruit quality parameters and guide robotic handling. Several deep learning approaches have further advanced ripeness classification. Imran et al.^13^ used VGG13, VGG16, and VGG19 with feature-fusion and SVM classification for chaunsa mango ripeness detection. Marshal et al.^14^ trained CNN models (VGG16, AlexNet) to distinguish mango varieties based on visual attributes. Earlier mango ripening studies also demonstrated the utility of image-based maturity assessment. Vélez-Rivera et al.^15^ developed a computer vision system for the classification of ‘Manila’ mangoes during ripening, showing that visual descriptors can support discrimination across ripening stages. Similarly, Mim et al.^16^ reported an automatic mango ripening-stage detection approach based on image analysis, further confirming the usefulness of external visual features for maturity classification. These studies strengthen the foundation for image-based mango ripeness assessment, while also highlighting that the success of purely visual methods depends on the cultivar and the distinctness of external ripening cues.

Beyond mangoes, CNN-based grading has been successfully applied to other crops. Alsirhani et al.^17^ classified date fruits using pretrained convolutional networks. Rodríguez et al.^18^ introduced a two-step deep learning framework for early-stage plum variety discrimination. For mango disease detection, Diana et al.^19^ used shape, colour, and texture descriptors with Artificial Bee Colony (ABC) optimization for feature selection. Studies in^20^ explored ML and transfer learning for papaya maturity classification, while Begum et al.^21^ evaluated advanced CNNs for tomato maturity, identifying ResNet152V2 as the most effective model.

Collectively, these works demonstrate that image processing and pretrained deep networks significantly enhance external grading performance across fruit types. However, reliance on external features alone can be problematic when visual cues are weak, inconsistent, or absent, as in NDMST mangoes.

### Grading with internal analysis

Internal indicators provide complementary insights into ripeness through changes in flesh firmness, internal colour, aroma, sugar accumulation, acidity, and starch-to-sugar conversion^4,22^. Several non-invasive sensing technologies enable internal quality assessment. FTIR, NIR, and Raman spectroscopy have been used to estimate attributes such as TSS and moisture content in tropical fruits and vegetables^23^, although NIR is limited by shallow penetration in fruits with thick rinds. MRI offers highly detailed internal imaging^24^, but its cost restricts routine agricultural deployment. Hyperspectral imaging (HSI) provides rich spectral information across dozens or hundreds of wavelengths^25,26^, but requires substantial computational resources and specialised instrumentation. Acoustic sensing^27^ assesses structural response via sound propagation but suffers from sensor–fruit impedance-matching challenges.

Broader analyses in^28^ highlight multiple descriptors—including colour, fluorescence, and spectral responses—captured through imaging and spectroscopy and used to train ML models for automated ripeness classification. Complementary findings in^29^ show that combining supervised and unsupervised ML methods can effectively predict mango ripeness, with feed-forward neural networks achieving the highest accuracy. While internal-analysis methods yield accurate biochemical insights, they typically involve high-cost equipment, slower acquisition, and operational constraints that hinder widespread adoption in commercial supply chains.

### Observations and motivation

*External analysis*: Image-based methods provide rapid, non-destructive assessment, but performance can be affected by environmental variations, bruising, transport damage, and fruit varieties that lack distinct visual ripening cues.

*Internal analysis*: Biochemical traits such as TSS and TA are strong predictors of ripeness, but technologies for measuring them non-destructively (e.g., NIR, HSI, MRI) are expensive and computationally intensive. For NDMST mangoes, whose peel colour remains largely unchanged throughout maturation, external visual cues alone are insufficient for reliable grading. Conversely, relying solely on internal analysis is impractical for most agricultural operations due to equipment cost and complexity. This gap motivates a hybrid approach.

The novelty of this work lies in proposing AFMG-DLFF, an *Integrative Extrinsic–Intrinsic Fusion* framework that combines RGB-based visual features with key biochemical indicators through deep learning and a feedforward cascade neural network (FFCNN). This multimodal strategy reduces subjectivity, enhances predictive accuracy, and enables scalable, automated maturity grading. By leveraging inexpensive RGB images enriched with biochemical context, AFMG-DLFF offers a non-destructive, and cost-effective alternative suitable for real-world smart farming environments.

## Methodology

The primary objective of this study is to develop AFMG-DLFF, a multimodal framework capable of accurately assessing the maturity stages of NDMST mangoes. The framework jointly analyses two complementary information sources: (i) external RGB images that reflect the fruit’s visual appearance, and (ii) internal biochemical traits that capture its physiological ripening behaviour. By integrating these modalities, AFMG-DLFF aims to overcome the limitations of conventional colour-based grading, which is unreliable for NDMST mangoes due to their visually uniform peel colour across ripening stages. The system ultimately classifies fruits into three maturity stages: unripe, semi-ripe, and ripe.

**Fig. 1:**
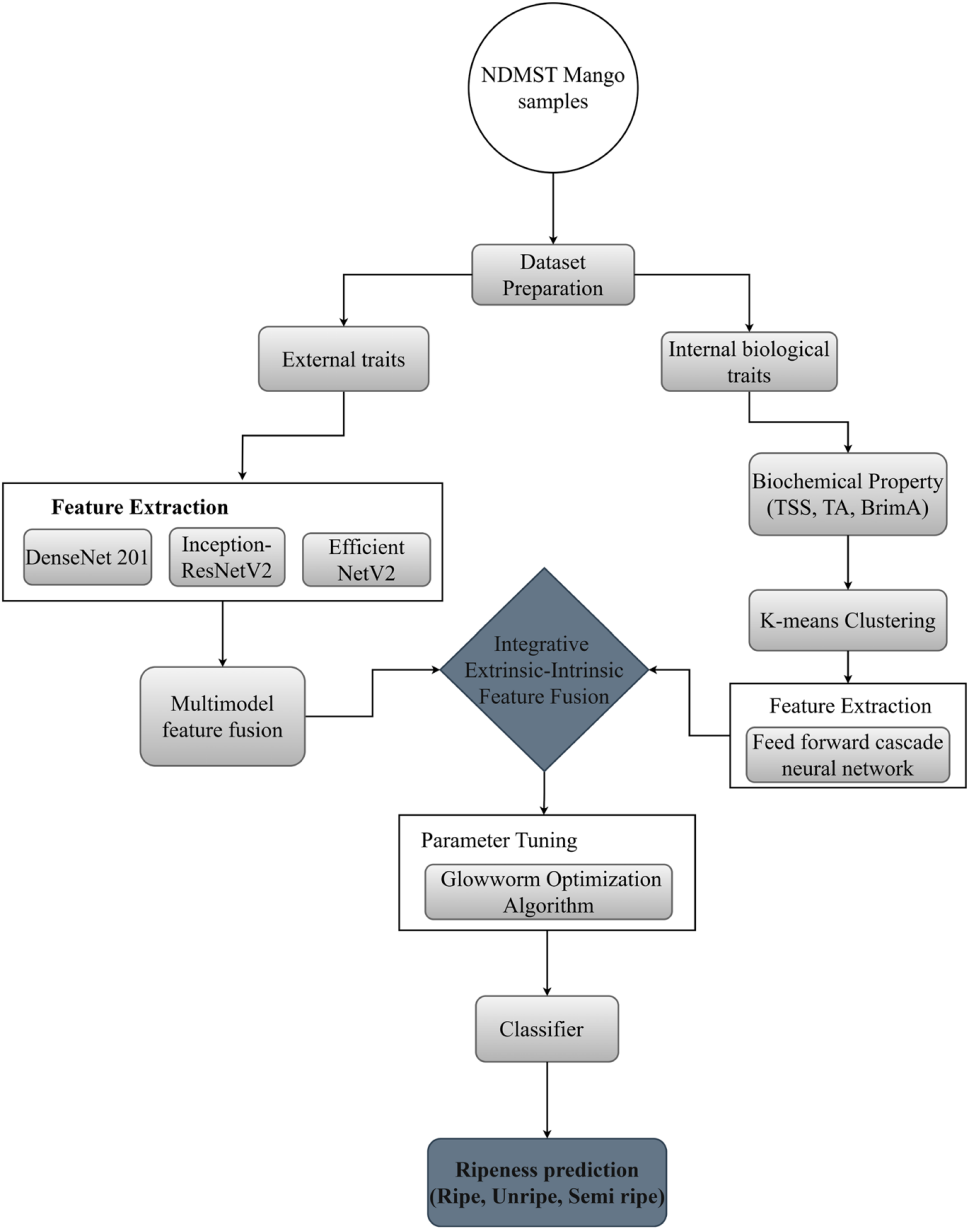
Workflow of the AFMG-DLFF system integrating image and biochemical features with GSO optimization for classification of NDMST mango maturity stages.

To anticipate the system’s performance in classifying NDMST mango ripeness stages, a multimodal dataset was constructed with two modules: an image module and a biochemical module. The image module contains RGB images of mangoes, while the biochemical module stores continuous internal attributes (TSS, TA, and BrimA) for the same fruits. Because the biochemical measurements do not have explicit categorical labels, *k*-means clustering is first applied to derive three maturity groups. These cluster-derived labels are then propagated to the corresponding images, forming a unified labelled dataset across both modalities. Visual features are subsequently extracted using pretrained CNNs, and internal features using a FFCNN. The AFMG-DLFF approach fuses these external and internal representations to create an integrative extrinsic–intrinsic feature vector. Initial fusion trials yielded modest gains, motivating the use of GSO for hyperparameter tuning and further performance improvement. The overall workflow of the proposed system is illustrated in Fig. 1.

### Dataset preparation

Data acquisition comprises two distinct phases: image collection for visual appearance analysis and biochemical measurement for internal quality assessment. In May 2023, a total of 120 NDMST mangoes were obtained. These fruits were harvested slightly earlier than the usual export maturity stage (85–95 days after fruit set, DAFS), specifically at 75–80 days after flowering, and subsequently sold to local vendors. Images and internal measurements were acquired to construct a comprehensive dataset for both visual and intrinsic trait analysis.

The NDMST cultivar was intentionally selected because it exhibits minimal external colour variation during ripening, providing a challenging test case for image-based ripeness assessment. Each fruit was assigned a unique identification code so that its RGB images and biochemical measurements could be matched, enabling the construction of real image–biochemical pairs for analysis and evaluation. These one-to-one pairs are preserved for the 600 real samples, whereas augmented training data later uses class-wise pairing between synthetic images and synthetic biochemical vectors. This work therefore serves as a proof-of-concept using a single-cultivar dataset collected under controlled conditions.

The experiments were conducted at the Department of Agricultural Marketing and Agribusiness, Sriperumbudur, Tamil Nadu, India. All experimental work, including mango sampling and destructive measurements, was performed at this site. Permission to conduct the study was formally obtained from the institution before initiating the work. No additional special permissions were required, as the study involved non-endangered fruit samples collected under controlled research conditions.

#### Experimental batch design

A total of 120 NDMST mangoes were observed over five consecutive ripening days (Day 1–Day 5). Each day, the same batch of 120 fruits was imaged and analysed for biochemical properties, producing 600 real image–biochemical pairs (120 fruits *×* 5 days).

#### Collection of images

For extrinsic dataset construction, images of 120 NDMST mangoes were acquired under controlled laboratory conditions. A 16 MP Android mobile camera positioned 15 cm above a white imaging table was used for capture. The camera was connected to a workstation running a 64-bit Windows operating system with 64 GB RAM, an NVIDIA Quadro P5000 GPU (16 GB memory), and an Intel Core i7 processor.

**Fig. 2:**
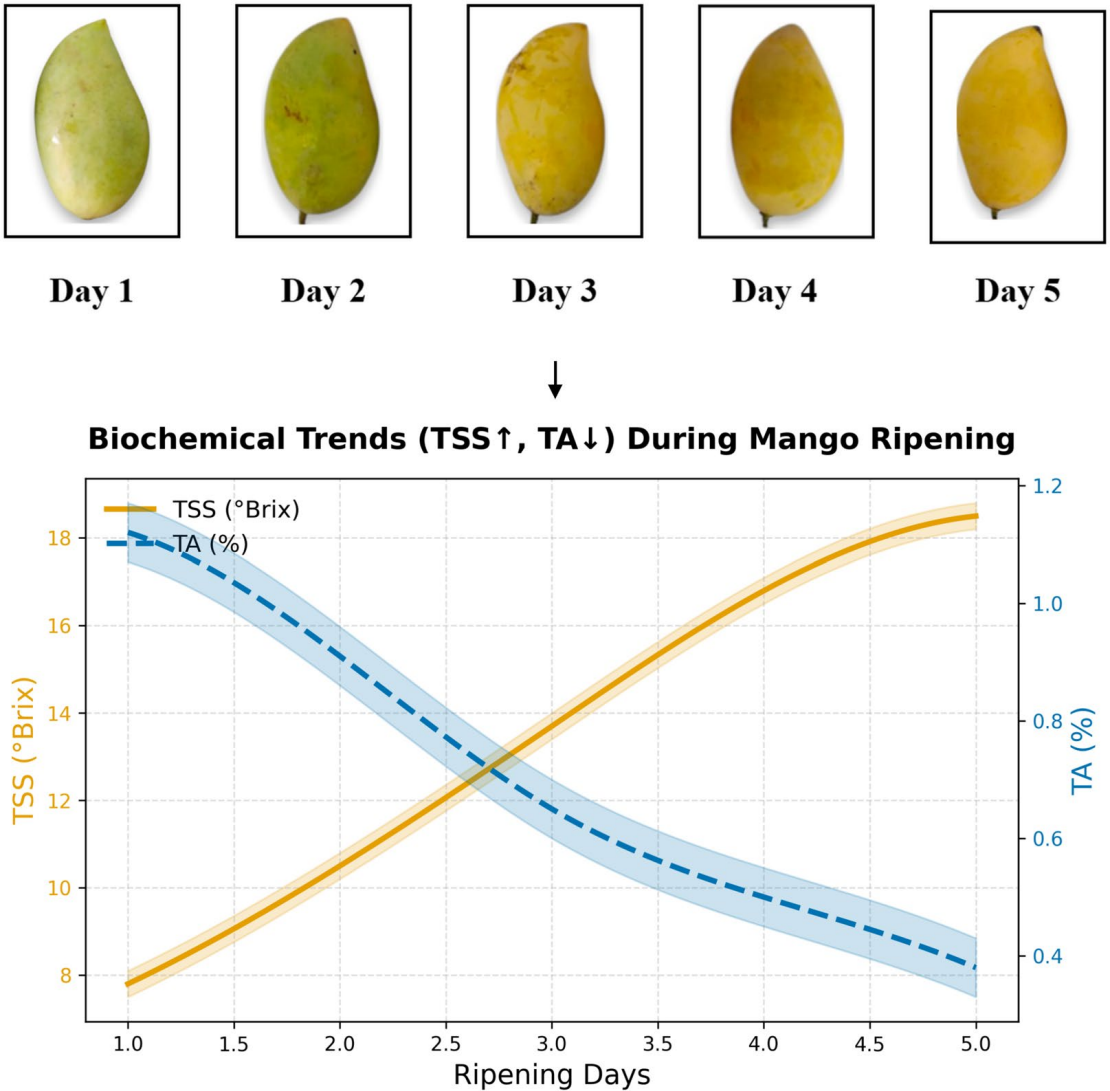
Ripening progression of NDMST mangoes from Day 1 to Day 5 showing minimal external colour transition across maturity stages and corresponding biochemical trends in TSS and TA during ripening.

Images were captured from multiple angles against a uniform white background to facilitate segmentation and enhance contrast. Based on observed ripening progression, mangoes were broadly categorised into three maturity stages: unripe (Day 1), semi-ripe (Days 2–3), and ripe (Days 4–5), as illustrated in Fig. 2. The figure also presents the corresponding biochemical variations, visually linking external appearance with internal compositional shifts during ripening.

#### Collection of biological properties

Fruit ripeness evaluation relies heavily on intrinsic biochemical characteristics that determine quality and taste. To construct the intrinsic dataset, the same 120 NDMST mangoes were analysed for TSS and TA over five ripening days, yielding 600 real measurements. TSS, measured using a digital refractometer, reflects the concentration of soluble sugars (expressed in °Brix) and represents the sweetness level of the fruit. TA, determined using titration and a pH meter, quantifies acidity and contributes to the sugar–acid balance influencing flavour. Typically, as ripening progresses, TSS increases due to starch-to-sugar conversion, while TA decreases, indicating a reduction in organic acid content and improved palatability.

The variations of these parameters across ripening days are shown in Fig. 2, demonstrating a consistent biochemical trend aligned with the visual ripening stages. Detailed dataset statistics are summarised in Table 1.

**Table 1:** Summary of the NDMST mango dataset showing the type of data collected, dataset characteristics, number of mango samples, and total sample count used for model development.

Dataset collection	Dataset characteristics	No. of mangoes	Total sample count
NDMST images	External traits	120	600
NDMST biological traits	TSS, TA	120	600

#### Calculation of BrimA

**Fig. 3:**
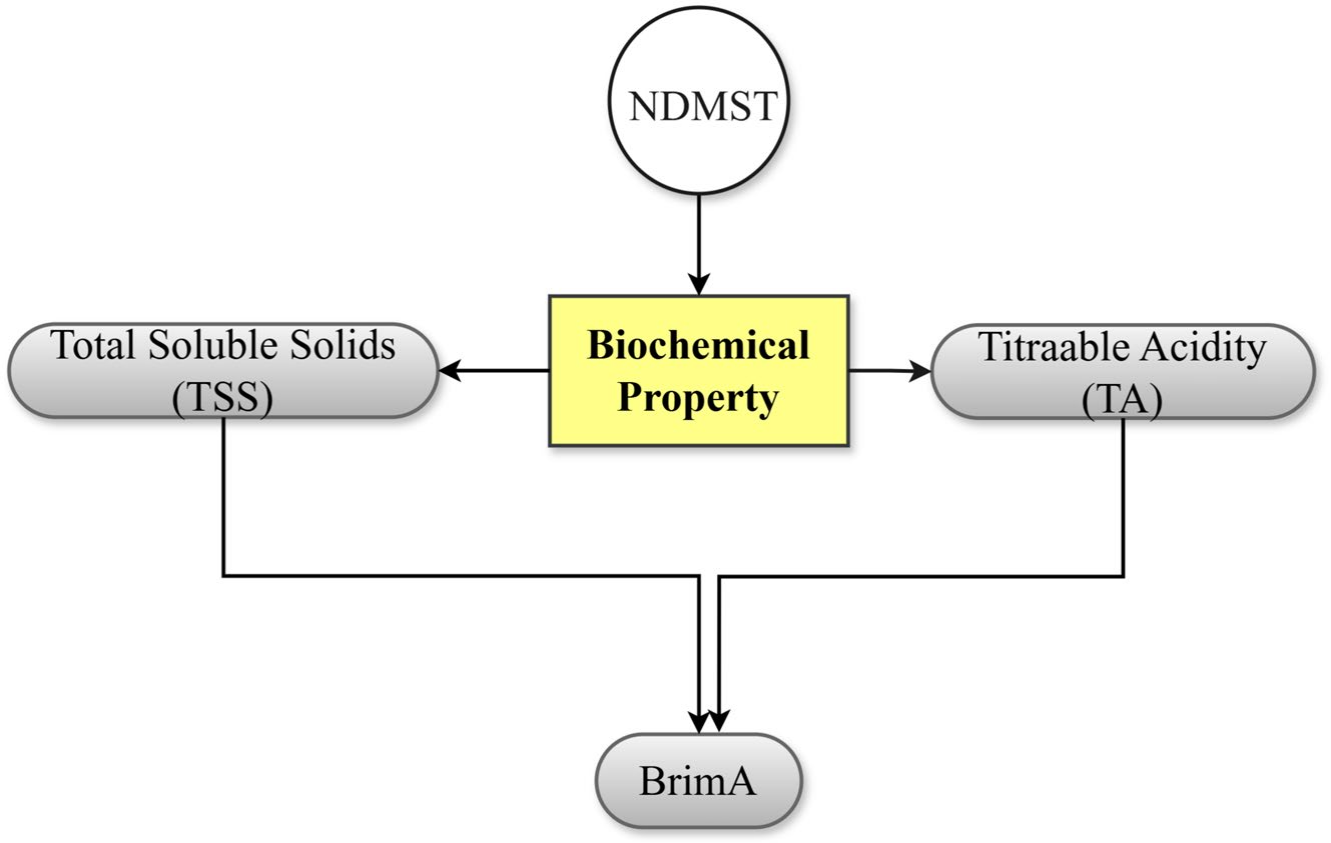
Biological traits of NDMST mangoes.

To complement the traditional Brix rating and incorporate the influence of acidity on perceived sweetness, the BrimA index was computed. BrimA extends beyond the simple TSS-to-TA ratio by penalising high acidity and has been recommended as a more perceptually aligned sweetness indicator, especially for fruits with pronounced acid content^30^. The internal traits used to assess fruit ripeness are illustrated in Fig. 3.

BrimA is defined as

1 $$\begin{aligned} \text {BrimA} = \text {TSS} - (k \times \text {TA}) \end{aligned}$$

where *k* is a cultivar-dependent weighting factor reflecting the typical sugar-to-acid ratio. For citrus fruits, *k* is often close to 5.0^30^; in this work, *k* was selected based on preliminary analysis of NDMST sweetness–acidity balance.

#### K-means clustering

The intrinsic biochemical dataset does not contain predefined ripeness labels. To derive ground truth categories for supervised learning, the three-dimensional biochemical feature vectors $[\text {TSS}, \text {TA}, \text {BrimA}]$ from all 600 real samples were clustered using the K-means algorithm with *K=3*, representing unripe, semi-ripe, and ripe groups^31^.

**Fig. 4:**
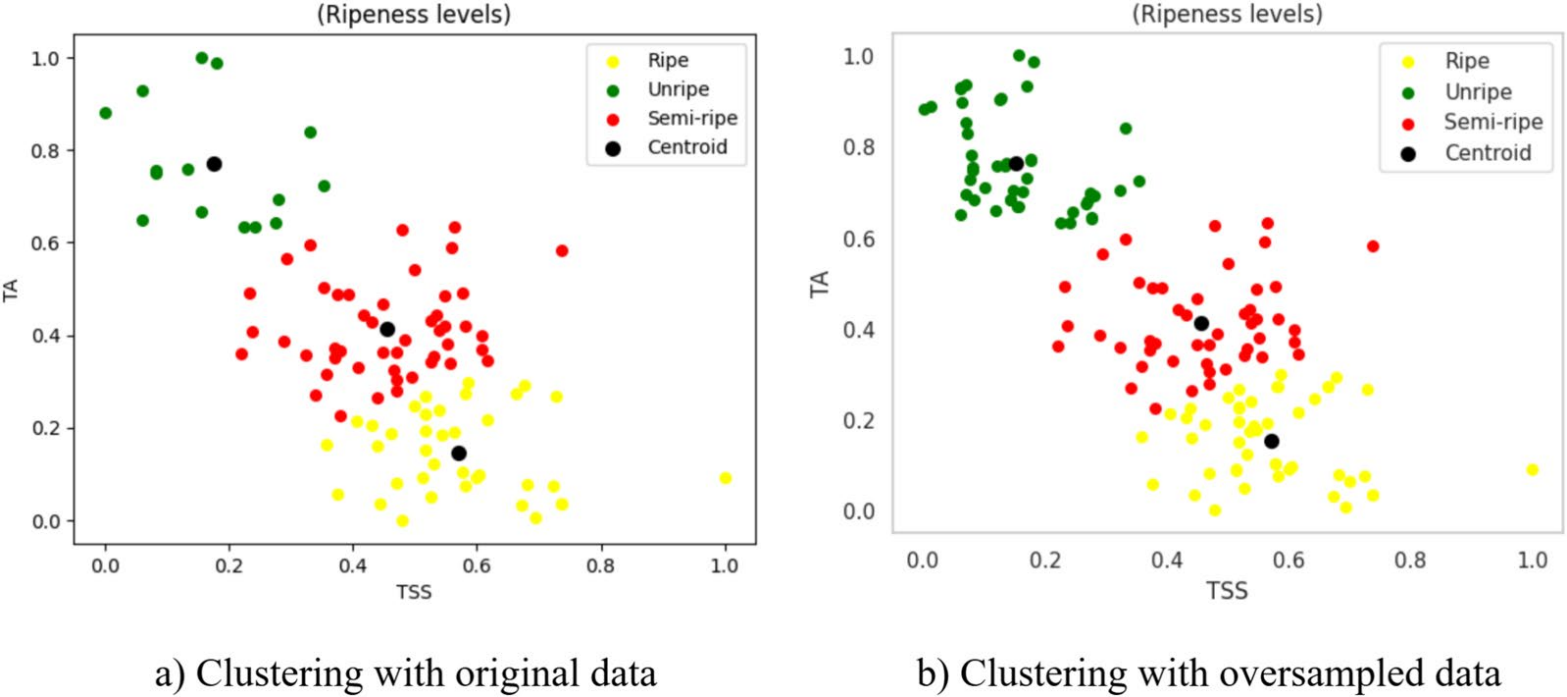
Clustering of mango maturity classes with respect to biochemical properties (TSS and TA) for (**a**) Original data and (**b**) Oversampled data illustrating clear separation among unripe, semi-ripe, and ripe stages.

K-means iteratively assigns each sample to the nearest centroid and updates centroids until convergence. The resulting three clusters, shown in Fig. 4a, exhibit distinct biochemical patterns: high TA and low TSS (Unripe), intermediate TSS and TA (semi-ripe), and high TSS with low TA (Ripe). These cluster-derived labels serve as the final ground-truth maturity classes used throughout this study and are propagated to the corresponding RGB images via the fruit ID.

Notably, Fig. 4a also reveals that the three ripeness clusters are not perfectly balanced in terms of sample counts. This class imbalance is handled *after* the train–test split by applying SMOTE exclusively to the training subset of the biochemical data, as detailed in “Pre-processing of biological traits” section and illustrated in Fig. 4b.

#### Labelling the data

The outcome of the *k*-means clustering procedure is a set of three classes that stratify the NDMST mango population into unripe, semi-ripe, and ripe stages based on TSS, TA, and BrimA. To provide an interpretable summary of these clusters, the BrimA index was normalised to the interval [0, 1] using min–max normalisation:

2 $$\begin{aligned} B_{\text {norm}} = \frac{\text {BrimA} - \min (\text {BrimA})}{\max (\text {BrimA}) - \min (\text {BrimA})}. \end{aligned}$$

Although the actual ripeness labels were obtained from *k*-means clustering in the full three-dimensional feature space $[\text {TSS}, \text {TA}, \text {BrimA}]$, the resulting clusters occupied distinct and non-overlapping intervals of $B_{\text {norm}}$, as summarised in Table 2. These intervals are reported as an interpretable characterisation of cluster properties, rather than as manually imposed threshold rules. The final class labels were used to align internal biological traits with the image dataset, forming the basis for supervised learning in the subsequent sections.

**Table 2:** Ripeness class labels for NDMST mangoes derived from K-means clustering of biochemical features, summarized using normalized BrimA ranges $B_{\text {norm}}$ and their associated biochemical patterns.

Biological attributes	$B_{\text {norm}}$ range	Ripening stages	Labels
Increase in TA, decrease in TSS	$0.0 \le B_{\text {norm}} \le 0.3$	Unripe	Class 0
Comparable TA and TSS (balanced acidity)	$0.4 \le B_{\text {norm}} \le 0.6$	semi-ripe	Class 2
Decrease in TA, increase in TSS	$0.7 \le B_{\text {norm}} \le 1.0$	Ripe	Class 1

### Dataset preprocessing

#### Image preprocessing

After acquiring the raw mango images, preprocessing was applied to enhance quality and standardise inputs. Each RGB image was resized to $224 \times 224 \times 3$ pixels, normalised, and subjected to basic geometric augmentation to improve generalisation. The dataset comprised 600 real RGB images captured from 120 NDMST mangoes over five ripening days. An 80:20 train–test split was adopted, stratified across the three maturity classes. The split was performed at the fruit-identity level so that all observations belonging to a given mango across ripening days were assigned to only one subset, thereby preventing train–test leakage from repeated measurements of the same fruit. Within the training portion, 10% of samples were reserved for validation, resulting in 432 training, 48 validation, and 120 independent test samples.

To mitigate overfitting caused by limited real data, a Conditional Generative Adversarial Network (CGAN) was employed for image augmentation. The CGAN was trained with a learning rate of 0.0002, batch size of 32, 100 epochs, and a latent vector of dimension 100. Augmentation was applied exclusively to the training images, expanding the training set to 2,450 samples while maintaining balanced class representation. For each synthetic image, the associated maturity label (unripe, semi-ripe, or ripe) was preserved, so that the CGAN effectively sampled from an empirical approximation of the class-conditional distribution $\hat{p}(I \mid y)$. These synthetic images were later used in multimodal training by pairing them with biochemical feature vectors drawn from the same maturity class (“Pre-processing of biological traits” section), ensuring that no cross-class mismatches occurred. Both validation and test subsets contained only real, unaugmented images to ensure unbiased evaluation. A concise summary of the dataset composition after preprocessing and augmentation is presented in Table 3.

**Table 3:** Summary of the augmented NDMST mango dataset after applying CGAN for image generation and SMOTE for biochemical data balancing, showing the number of real and synthetic samples used across training, validation, and testing subsets.

Dataset Type	Subset	Data Source/Method	Real samples	Synthetic samples	Total samples	Purpose
Image dataset (RGB)	Training (70%)	CGAN-based augmentation	432	2,018	2,450	Model training
	Validation (10% of training)	Real (10% of training)	48	–	48	Early stopping, overfitting control
	Testing (20%)	Real (independent)	120	–	120	Final model evaluation
Biochemical dataset (TSS, TA)	Training (70%)	SMOTE oversampling	432	2,018	2,450	Model training
	Validation (10% of training)	Real (10% of training)	48	–	48	Early stopping, overfitting control
	Testing (20%)	Real (independent)	120	–	120	Final model evaluation

#### Pre-processing of biological traits

Biochemical attributes corresponding to the same 120 NDMST mangoes were obtained over five ripening days, yielding 600 real image–biochemical pairs. For each fruit and each day, TSS and TA were measured, and the BrimA index was computed, forming a three-dimensional biochemical feature vector $x = [\text {TSS}, \text {TA}, \text {BrimA}]$. Prior to analysis, these features were normalised and rounded to ensure consistency across observations. The biochemical dataset followed the same 80:20 train–test split used for images (432 training, 48 validation, 120 test). The same fruit-identity-based partition was preserved for the biochemical branch to maintain alignment between modalities and avoid repeated-fruit leakage across subsets.

To correct class imbalance within the training subset, SMOTE was applied only to the 432 training samples. SMOTE generated synthetic feature vectors by interpolating between minority-class neighbours within each maturity class, producing a balanced biochemical training set of 2,450 samples and approximating the class-conditional distribution $\hat{p}(x \mid y)$, where $x = [\text {TSS}, \text {TA}, \text {BrimA}]$. Validation and test subsets were retained as real, unaugmented data to preserve independence during evaluation.

For multimodal training, CGAN-generated images and SMOTE-generated biochemical vectors were paired at the class level. Specifically, for each maturity class $y \in \{\text {unripe}, \text {semi-ripe}, \text {ripe}\}$, synthetic RGB images and synthetic biochemical vectors were randomly matched *within* that class. This procedure is equivalent to sampling from the product distribution $\hat{p}(I, x \mid y) \approx \hat{p}(I \mid y)\,\hat{p}(x \mid y)$ under a conditional-independence assumption between image appearance and biochemical traits given the ripening stage. Importantly, no image was ever paired with a biochemical vector from a different class, and all validation and test results were computed using only real image–biochemical pairs. This design ensures that augmentation enriches intra-class variability during training while final performance is always measured on physiologically consistent, real-world samples.

It is important to note that preliminary trials conducted using only the 600 real image–biochemical pairs resulted in rapid overfitting of the deep visual encoders and unstable convergence in the multimodal classifier. This behaviour was primarily due to the extremely limited RGB dataset and the class imbalance revealed by k-means clustering. Consequently, SMOTE and CGAN augmentation were introduced not to inflate performance but to stabilise optimisation by increasing intra-class variability during training. Importantly, all validation and test evaluations are performed exclusively on real, unaugmented samples, ensuring that augmentation does not affect the reported generalisation accuracy.

### Feature extraction

#### Intrinsic feature extraction

Intrinsic features were extracted from the biochemical traits using a FFCNN. The model was trained to learn a compact latent representation of the biochemical vectors $x = [\text {TSS}, \text {TA}, \text {BrimA}]$ and their associated ripeness labels derived from *k*-means clustering. A dense bottleneck layer with 512 units served as the intrinsic feature layer. Using the 2,450 training samples (real + SMOTE-augmented), the FFCNN produced a feature matrix $I_{fv} \in {\mathbb {R}}^{2450 \times 512}$, where each row corresponds to a 512-dimensional intrinsic representation $I_{fv} = [i_{fv1}, i_{fv2}, \ldots , i_{fv512}]$. Algorithm 1 summarises the complete procedure for intrinsic feature extraction.

**Algorithm 1:**
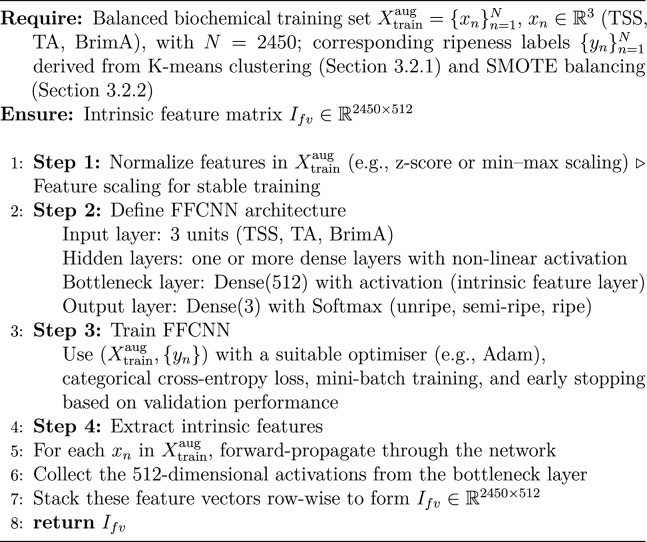
Intrinsic feature extraction.

#### Extrinsic feature extraction

To extract discriminative visual features from the RGB images of NDMST mangoes, three state-of-the-art pretrained convolutional neural networks—DenseNet201, Inception–ResNetV2, and EfficientNetV2—were employed. These models were selected because they capture complementary representations: DenseNet201 emphasises feature reuse and boundary details, Inception–ResNetV2 captures multi-scale structures using parallel convolutional branches, and EfficientNetV2 provides computationally efficient deep features through compound scaling and Fused-MBConv blocks. Together, they provide a diverse and robust feature set suitable for downstream multimodal fusion. Since training deep CNNs from scratch requires large datasets, transfer learning was used. Each RGB image $N_i$ was resized to $224 \times 224 \times 3$ and passed through the pretrained networks with their classification layers removed. For each model, the output of the final convolutional block was processed using Global Average Pooling (GAP) to obtain a fixed-length descriptor. The GAP output was then flattened to form a 512-dimensional feature vector for each image. *DenseNet201*: DenseNet201 consists of densely connected convolutional layers, where each layer receives the feature maps of all preceding layers. This architecture promotes strong feature propagation and mitigates vanishing gradients. The output of the final dense block, after GAP, yields a 512-dimensional feature vector capturing spatial and textural cues relevant to mango surface characteristics. *Inception–ResNetV2*: Inception–ResNetV2 combines Inception modules with residual connections to extract multi-scale features while preserving stable gradient flow. The network processes the input through a stem block, several Inception–ResNet modules, and reduction blocks. GAP at the network’s output produces a compact 512-dimensional descriptor encoding multi-scale visual patterns such as colour gradients and fine-grained texture variations. *EfficientNetV2*: EfficientNetV2 leverages compound scaling and Fused-MBConv blocks to achieve efficient feature extraction with fewer parameters. Its architecture incorporates batch-normalized convolutions, depthwise MBConv layers, and squeeze-and-excitation (SE) attention mechanisms. After passing the augmented mango image $N_i$ through the convolutional backbone and applying GAP, a 512-dimensional feature vector is obtained, capturing both global and local visual structures. For each image, the three resulting feature vectors:${\textbf{D}}_{fv}$, $\textbf{IR}_{fv}$, and $\textbf{E}_{fv}$ represent complementary views of the fruit’s external appearance. These vectors are concatenated in “Multimodel feature fusion” section to form the multimodel fused feature representation.

### Multimodel feature fusion

The goal of multimodel feature fusion is to combine the complementary features extracted by DenseNet201, Inception–ResNetV2, and EfficientNetV2 from the NDMST image dataset into a single unified representation for ripeness classification. For each input image $N_i$, each pretrained network produces a 512-dimensional feature vector. These are denoted as $\textbf{D}_{fv}$, $\textbf{IR}_{fv}$, and $\textbf{E}_{fv}$ for DenseNet201, Inception–ResNetV2, and EfficientNetV2, respectively, and can be written as

3 $$\begin{aligned} \textbf{D}_{fv}= & [d_{fv1}, d_{fv2}, \ldots , d_{fvi}, \ldots , d_{fv512}], \end{aligned}$$

4 $$\begin{aligned} \textbf{IR}_{fv}= & [ir_{fv1}, ir_{fv2}, \ldots , ir_{fvj}, \ldots , ir_{fv512}], \end{aligned}$$

5 $$\begin{aligned} \textbf{E}_{fv}= & [e_{fv1}, e_{fv2}, \ldots , e_{fvk}, \ldots , e_{fv512}], \end{aligned}$$

for DenseNet201, Inception–ResNetV2, and EfficientNetV2, respectively.

To form a unified visual feature representation, a concatenation-based multimodel fusion strategy is employed. The three 512-dimensional feature vectors are stacked to construct a single 1536-dimensional feature vector:

6 $$\begin{aligned} \textbf{F}_{fv} = \textbf{D}_{fv} \oplus \textbf{IR}_{fv} \oplus \textbf{E}_{fv}, \end{aligned}$$

where *⊕* denotes vector concatenation.

**Fig. 5:**
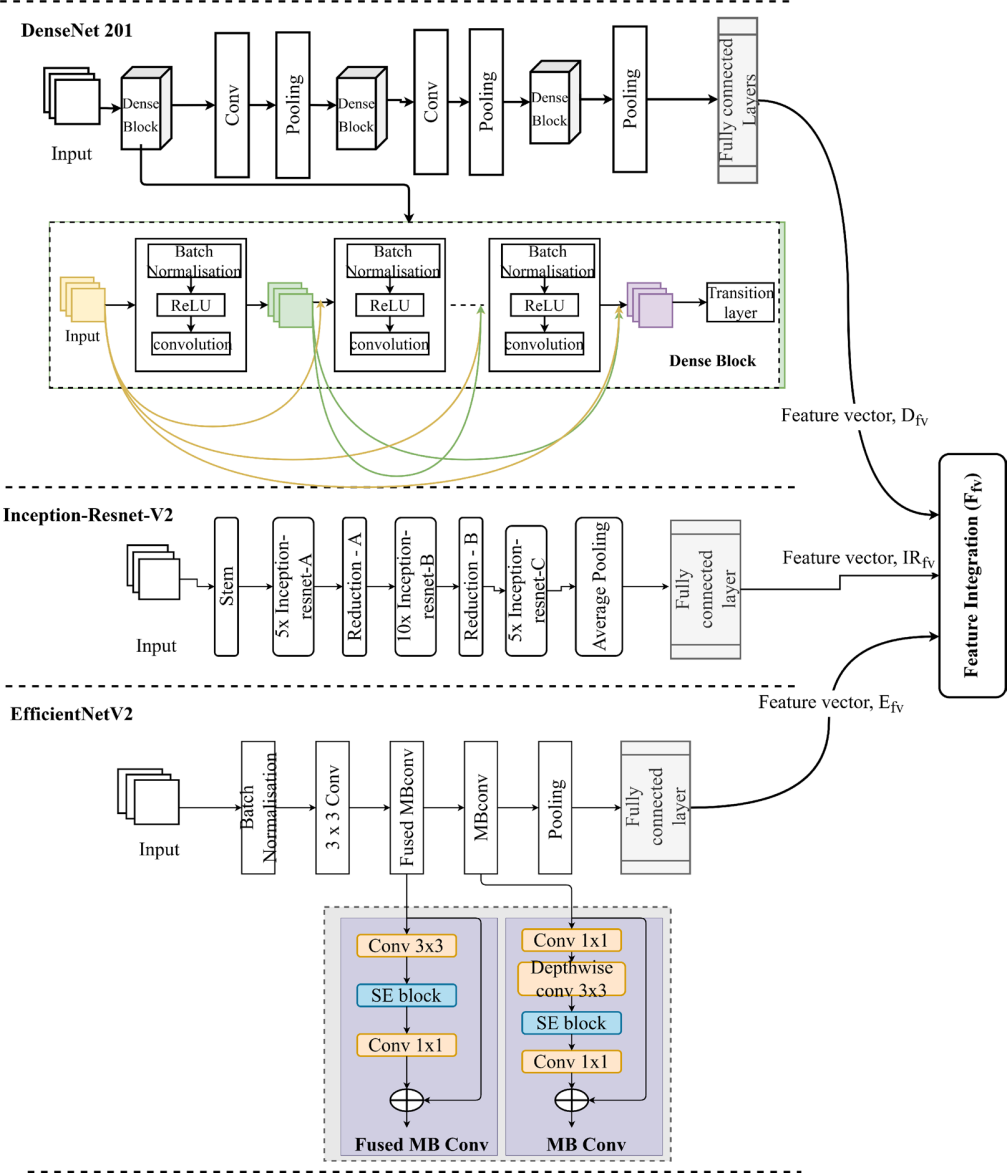
Multimodel feature fusion architecture combining features from DenseNet201, Inception-ResNetV2, and EfficientNetV2 to generate a unified representation for NDMST mango maturity classification.

The resulting multimodel fusion matrix $\textbf{F}_{fv} \in {\mathbb {R}}^{2450 \times 1536}$ contains 2,450 fused feature vectors (one per augmented training image), each of length 1536. By combining the strengths of the three pretrained models feature reuse in DenseNet201, multi-scale representation in Inception–ResNetV2, and efficient deep feature extraction in EfficientNetV2 this fused representation captures a richer set of external characteristics than any single model alone. Algorithm 2 outlines the multimodel feature fusion procedure, and Fig. 5 visually illustrates the integration of features from the three CNN backbones.

**Algorithm 2:**
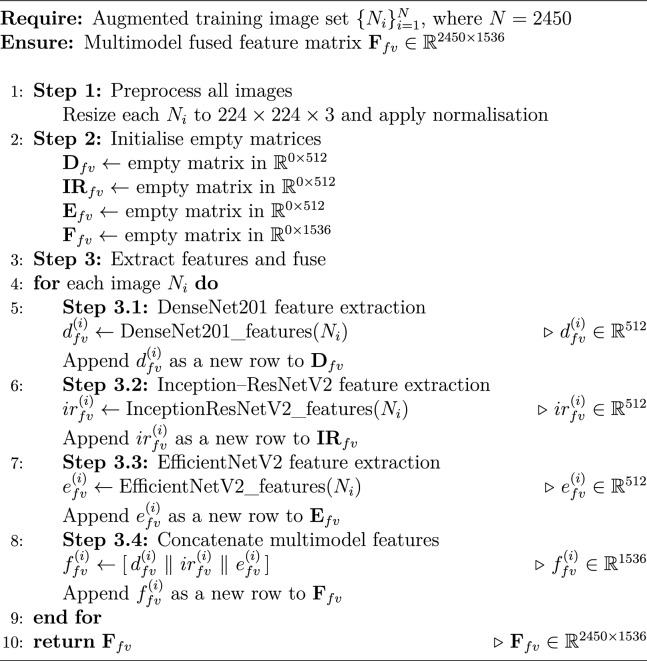
Multimodel feature fusion.

### Integrative Extrinsic–Intrinsic Fusion

The purpose of the integrative extrinsic–intrinsic fusion stage is to combine the complementary information obtained from RGB images (extrinsic cues) and biochemical measurements (intrinsic cues) into a unified multimodal representation suitable for final maturity classification. The dimensionality of the fused representation reflects the combined visual and physiological characteristics of each NDMST mango.

Intrinsic features were first extracted from the biochemical attributes using a FFCNN, yielding a feature matrix $I_{fv} \in {\mathbb {R}}^{2450 \times 512}$. In parallel, extrinsic features were extracted from the three pretrained deep learning models (DenseNet201, Inception–ResNetV2, EfficientNetV2) and concatenated to form the multimodel visual feature matrix $F_{fv} \in {\mathbb {R}}^{2450 \times 1536}$.

**Assumptions underlying feature fusion.** The proposed fusion strategy is based on four assumptions. First, the three pretrained visual encoders are assumed to provide complementary rather than redundant information; therefore, their outputs are concatenated to preserve the full joint representation without manual weighting. Second, for the 600 real samples, RGB images and biochemical measurements are one-to-one matched through fruit identity and ripening day. Third, for augmented training data, CGAN-generated images and SMOTE-generated biochemical vectors are paired only within the same ripening class, which corresponds to an approximate conditional-independence assumption between external appearance and biochemical traits given the maturity label, i.e., $\hat{p}(I, x \mid y) \approx \hat{p}(I \mid y)\,\hat{p}(x \mid y)$. Fourth, no cross-class pairings are introduced, and all validation and test evaluations are performed exclusively on real image–biochemical pairs to ensure physiologically meaningful assessment.

After class-wise pairing, the intrinsic and extrinsic feature vectors were concatenated to form the final fused representation $\delta = D_{fv} \oplus IR_{fv} \oplus E_{fv} \oplus I_{fv}$, where $\delta \in {\mathbb {R}}^{2450 \times 2048}$ and each modality contributes a 512-dimensional subvector. This unified feature vector captures both visual and physiological dimensions of fruit maturity and serves as the input to the downstream classifier.

Although CGAN and SMOTE augmentation enlarged the training distribution, they did not alter the evaluation protocol, because validation and test performance was always computed using only real image–biochemical pairs.

**Algorithm 3:**
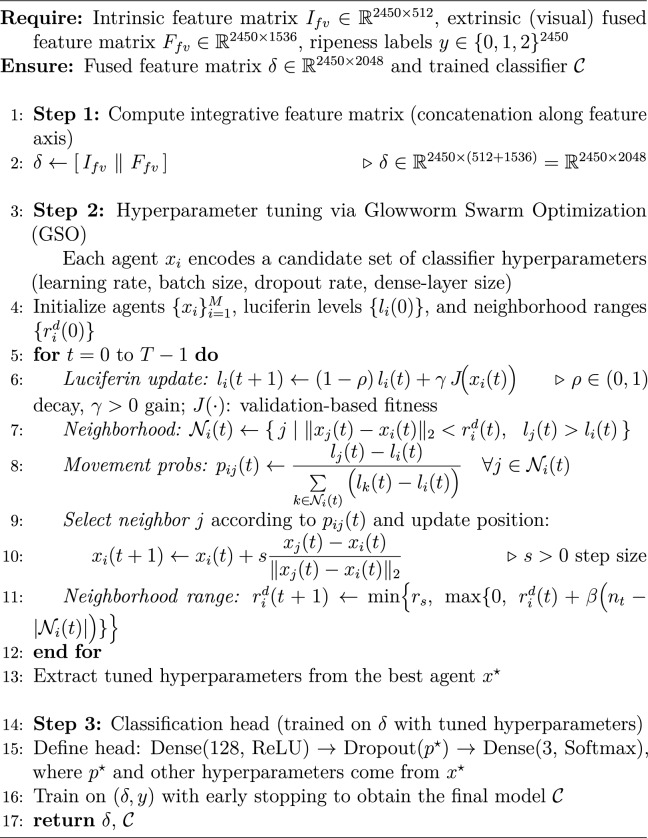
Integrative extrinsic–intrinsic fusion and classification.

### Hyperparameter optimization

To further enhance the performance of the AFMG-DLFF framework after multimodal feature fusion, hyperparameter tuning was performed using the Glowworm Swarm Optimization (GSO) algorithm. GSO, inspired by the collective behaviour of glowworms, is suitable for continuous and multimodal search spaces^32^. In the present study, GSO was adopted as the hyperparameter optimization strategy because its adaptive neighbourhood search offers a practical balance between exploration and exploitation when tuning the classifier operating on the fused feature representation. Other optimization approaches, including PSO, GA, and Bayesian optimization, are also viable alternatives; however, a systematic comparative study across optimization algorithms was outside the scope of the present work.

In this study, GSO was applied to tune key hyperparameters of the classifier operating on the fused representation *δ*. The search space included the learning rate (0.0001–0.001), batch size (16–64), dropout rate (0.2–0.5), and dense-layer size (64–256). Each glowworm encoded a candidate hyperparameter vector, and its luciferin value was updated based on validation accuracy and loss, allowing the swarm to iteratively migrate toward regions of improved model performance. The optimisation process converged to the following hyperparameters: learning rate = 0.0003, batch size = 32, dropout = 0.3, and dense-layer size = 128. These tuned settings provided stable training dynamics and the highest validation accuracy within the adopted optimization framework, and were subsequently used for the final maturity-stage classification of NDMST mangoes.

## Empirical findings and discussions

To assess the efficacy of the proposed AFMG-DLFF method for grading the maturity stages of NDMST mangoes, a multimodal feature-fusion approach was implemented that integrates external image characteristics with internal biochemical traits. All experiments were conducted on a workstation running a 64-bit Windows operating system, equipped with 64 GB of RAM, an NVIDIA Quadro P5000 GPU (16 GB memory), and an Intel i7 processor. The models were developed using Python with Keras, TensorFlow, and OpenCV libraries. The following sections present the experimental results and discuss the effectiveness of the AFMG-DLFF framework in comparison with existing state-of-the-art methods.

### Evaluation results of internal biological traits

Based on the labelling scheme defined in “Labelling the data” section, the clustering process provided the foundation for transitioning from an unsupervised to a supervised model. The ripeness stages were clearly distinguishable across three categories (Table 2):

- *Unripe (Class 0)*: fruits with high acidity (TA) and low sweetness (TSS), with $B_{\text {norm}}$ in the range 0.0–0.3.
- *Ripe (Class 1)*: fruits with high sweetness (TSS) and reduced acidity (TA), with $B_{\text {norm}}$ in the range 0.7–1.0.
- *Semi-ripe (Class 2)*: fruits with a balanced sugar-to-acid ratio (TSS/TA), clustered around $B_{\text {norm}}$ = 0.4–0.6.

Following this classification, FFCNN models were trained using the internal biological traits. The extracted feature vectors captured discriminative patterns from the dense bottleneck layer, enabling effective assessment of mango ripeness based solely on intrinsic properties.

### Evaluation results of external analysis

Three pretrained deep learning models—DenseNet201, Inception–ResNetV2, and EfficientNetV2—were utilized for feature extraction from NDMST images. These models were individually evaluated within the AFMG-DLFF framework to quantify their standalone performance, which is crucial for subsequent model selection and fusion. In the comparative evaluation, DenseNet201 achieved an accuracy of ${D}_{acc} = 85.53\%$, demonstrating robust performance. Inception–ResNetV2 achieved ${IR}_{acc} = 82.34\%$, effectively capturing both fine-grained and global features. EfficientNetV2 achieved the highest single-model accuracy of ${Eff}_{acc} = 88.16\%$. Its compound-scaling design allowed efficient capture of both fine and coarse image patterns. Transfer learning enabled all three models to leverage prior knowledge from large-scale datasets, providing valuable representations despite the limited training data. Overall, these pretrained networks demonstrated strong capability in image-based feature extraction tasks for NDMST maturity classification.

#### Multimodel feature fusion

The multimodel feature-fusion (MFF) approach combined the feature representations from DenseNet201, Inception–ResNetV2, and EfficientNetV2, leveraging their complementary strengths. DenseNet201 contributed dense feature connectivity, Inception–ResNetV2 captured multi-scale spatial patterns, and EfficientNetV2 efficiently encoded complex structural features. The fusion of these representations produced a unified, high-dimensional feature vector that enriched the information available for ripeness grading. The ensemble MFF model achieved an accuracy of $MFF_{acc} = 88.73\%$, demonstrating the effectiveness of integrating diverse pretrained features. This improvement over the individual models underscores the advantage of multimodel fusion in enhancing classification accuracy for NDMST ripeness prediction.

#### Integrative extrinsic-intrinsic fusion and the effect of GSO optimization

The integrative extrinsic–intrinsic fusion strategy combined multimodel image features extracted through the MFF process with the internal biochemical attributes of the NDMST mangoes. This integration generated a comprehensive feature representation that simultaneously captured external visual traits and intrinsic biochemical indicators. The MFF model alone achieved an accuracy of *88.73%*, while inclusion of biochemical traits enhanced the overall accuracy to *94.28%* (Table 4). These results demonstrate that intrinsic parameters such as TSS and TA provide complementary discriminative cues that strengthen visual classification and significantly improve maturity prediction.

**Table 4:** Performance comparison of individual deep learning models and the multimodel feature-fusion (MFF) approach before GSO optimization, showing classification accuracy for NDMST mango maturity prediction.

Model	Accuracy (%)
AFMG-DLFF	94.28
Multimodel feature fusion	88.73
DenseNet201	85.53
InceptionResNetV2	82.34
EfficientNetV2	88.16

To further refine model performance, GSO was employed for hyperparameter tuning. As detailed in “Hyperparameter optimization” section, GSO was adopted in this study as the hyperparameter optimization strategy for the fused-feature classifier because its adaptive neighbourhood search is well suited to the present tuning problem. GSO optimised four key parameters of the classifier—learning rate (0.0001–0.001), batch size (16–64), dropout rate (0.2–0.5), and dense-layer dimension (64–256)—and identified the optimal configuration (learning rate = 0.0003, batch size = 32, dropout = 0.3, dense layer = 128). Following GSO optimization, the AFMG-DLFF model achieved an improved accuracy of *97.86%* (Table 5). Although the numerical gain of approximately 3% appears moderate, it reflects enhanced training stability and stronger generalisation.

*Note on augmentation ablation.* A real-only ablation (training without CGAN or SMOTE augmentation) was not reported because, with only 600 real image–biochemical pairs and the class imbalance revealed by *k*-means clustering, the deep visual encoders rapidly overfitted and the multimodal classifier failed to converge reliably. Augmentation was therefore a necessary stabilisation step rather than a means to inflate performance. Importantly, all validation and test results are computed strictly on real, unaugmented samples, ensuring that the reported accuracy reflects genuine generalisation.

**Table 5:** Performance comparison of individual deep learning models and the multimodel feature-fusion (MFF) approach *after* GSO optimization, showing improved classification accuracy for NDMST mango maturity prediction.

Model	Accuracy (%)
AFMG-DLFF	97.86
Multimodel feature fusion	91.52
DenseNet201	87.32
InceptionResNetV2	85.74
EfficientNetV2	80.69

### Performance metrics

The performance of the proposed AFMG-DLFF framework was quantitatively evaluated using accuracy, precision, recall, and F1-score. For each ripening class $c \in \{\text {unripe}, \text {semi-ripe}, \text {ripe}\}$, we compute the number of true positives ($tp_c$), true negatives ($tn_c$), false positives ($fp_c$), and false negatives ($fn_c$), where:

- True Positive ($tp_c$): number of samples belonging to class *c* that are correctly predicted as class *c*;
- True Negative ($tn_c$): number of samples not belonging to class *c* that are correctly predicted as not class *c*;
- False Positive ($fp_c$): number of samples not belonging to class *c* that are incorrectly predicted as class *c*;
- False Negative ($fn_c$): number of samples belonging to class *c* that are incorrectly predicted as not class *c*.

The evaluation metrics are then derived per class using:

7 $$\begin{aligned} \text {Accuracy}_c= & \frac{tp_c+tn_c}{tp_c+tn_c+fp_c+fn_c}, \end{aligned}$$

8 $$\begin{aligned} \text {Precision}_c= & \frac{tp_c}{tp_c+fp_c}, \end{aligned}$$

9 $$\begin{aligned} \text {Recall}_c= & \frac{tp_c}{tp_c+fn_c}, \end{aligned}$$

10 $$\begin{aligned} \text {F1-score}_c= & \frac{2 \times \text {Precision}_c \times \text {Recall}_c}{\text {Precision}_c + \text {Recall}_c}. \end{aligned}$$

**Table 6:** Performance metrics of the proposed AFMG-DLFF model before and after GSO optimization, presenting precision, recall, F1-score, and accuracy for each ripening class of NDMST mangoes.

Model	Class	Precision	Recall	F1-score	Accuracy
AFMG-DLFF (before GSO)	Unripe	0.98	0.98	0.98	0.97
	semi-ripe	0.97	0.93	0.95	0.95
	Ripe	0.98	0.99	0.98	0.98
AFMG-DLFF (after GSO)	Unripe	0.99	0.99	0.99	0.99
	semi-ripe	0.98	0.97	0.98	0.98
	Ripe	0.99	0.98	0.98	0.98

**Fig. 6:**
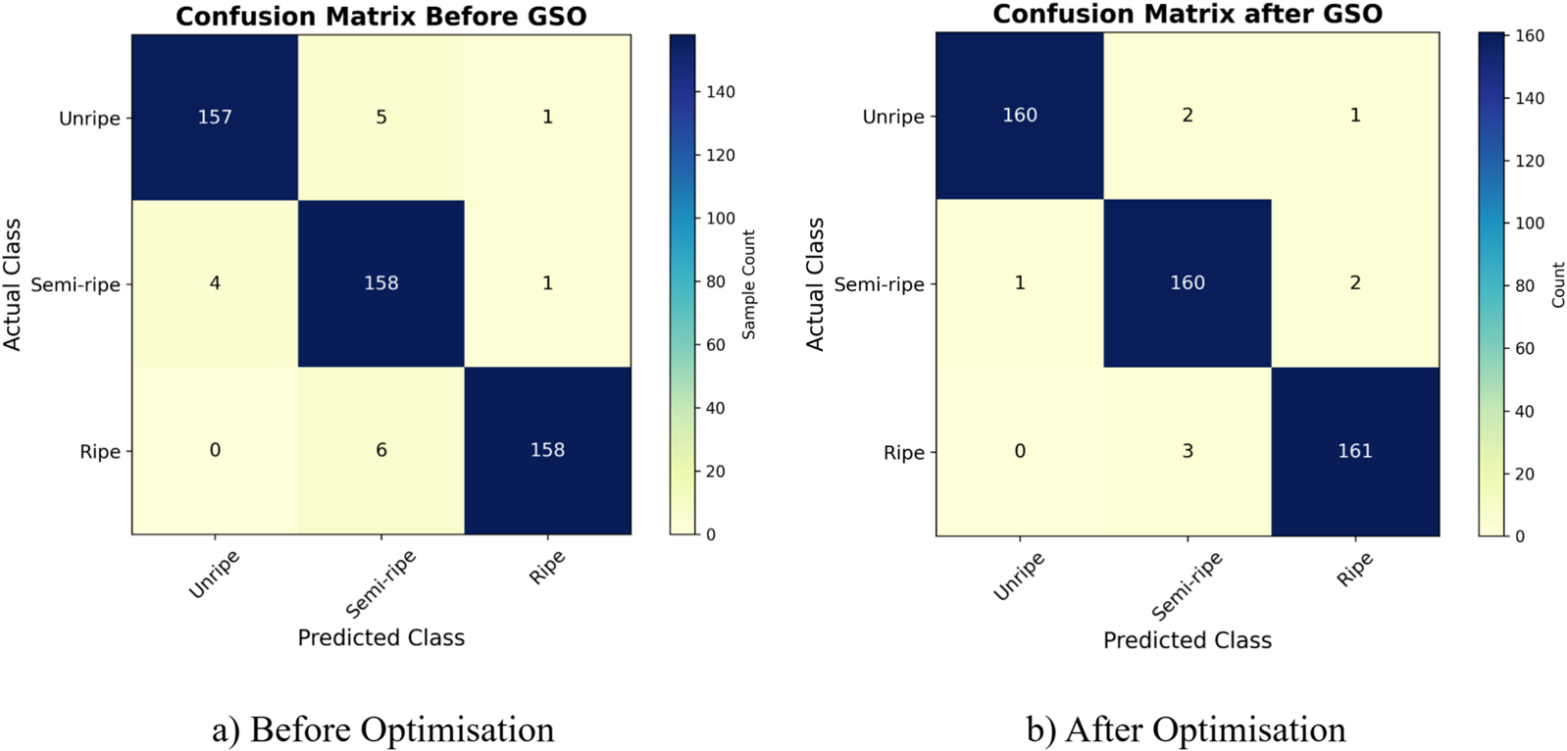
Confusion matrices of the proposed AFMG-DLFF model showing class-wise prediction performance (**a**) before optimization and (**b**) after optimization for unripe, semi-ripe, and ripe NDMST mangoes.

Table 6 presents the class-wise precision, recall, F1-score, and accuracy of the AFMG-DLFF framework before and after optimization. The optimised model consistently achieved precision, recall, and F1-scores above 0.97, confirming balanced predictive performance across all maturity classes. The confusion matrices in Fig. 6 further demonstrate clear separation between unripe, semi-ripe, and ripe categories, with only minor boundary overlaps.

The inclusion of both external and internal features markedly enhanced classification capability, while GSO-based optimization contributed an additional *∼*3% overall accuracy gain and reduced class-wise misclassification (Table 7). Although incremental, this improvement is meaningful for precision-dependent grading applications. With larger and multi-season datasets, further optimization is expected to yield greater benefits by exploring a wider hyperparameter space. Table 7 summarises the comparison of class-wise accuracy and misclassification before and after optimization, showing that misclassification decreased from 2–3% to about 1% across all ripening stages. Together, these results confirm that fusing extrinsic and intrinsic attributes, supported by GSO-based hyperparameter tuning, produces a high-performing and robust classifier for non-destructive maturity assessment of NDMST mangoes.

**Table 7:** Outcome of each ripening class within the proposed AFMG-DLFF model, showing accuracy and misclassification rates before and after GSO optimization for unripe, semi-ripe, and ripe NDMST mangoes.

Class	Before optimization		After optimization	
	Accuracy	Misclassification	Accuracy	Misclassification
Unripe	0.98	0.02	0.99	0.01
Semi-ripe	0.97	0.03	0.98	0.02
Ripe	0.98	0.02	0.99	0.01

**Fig. 7:**
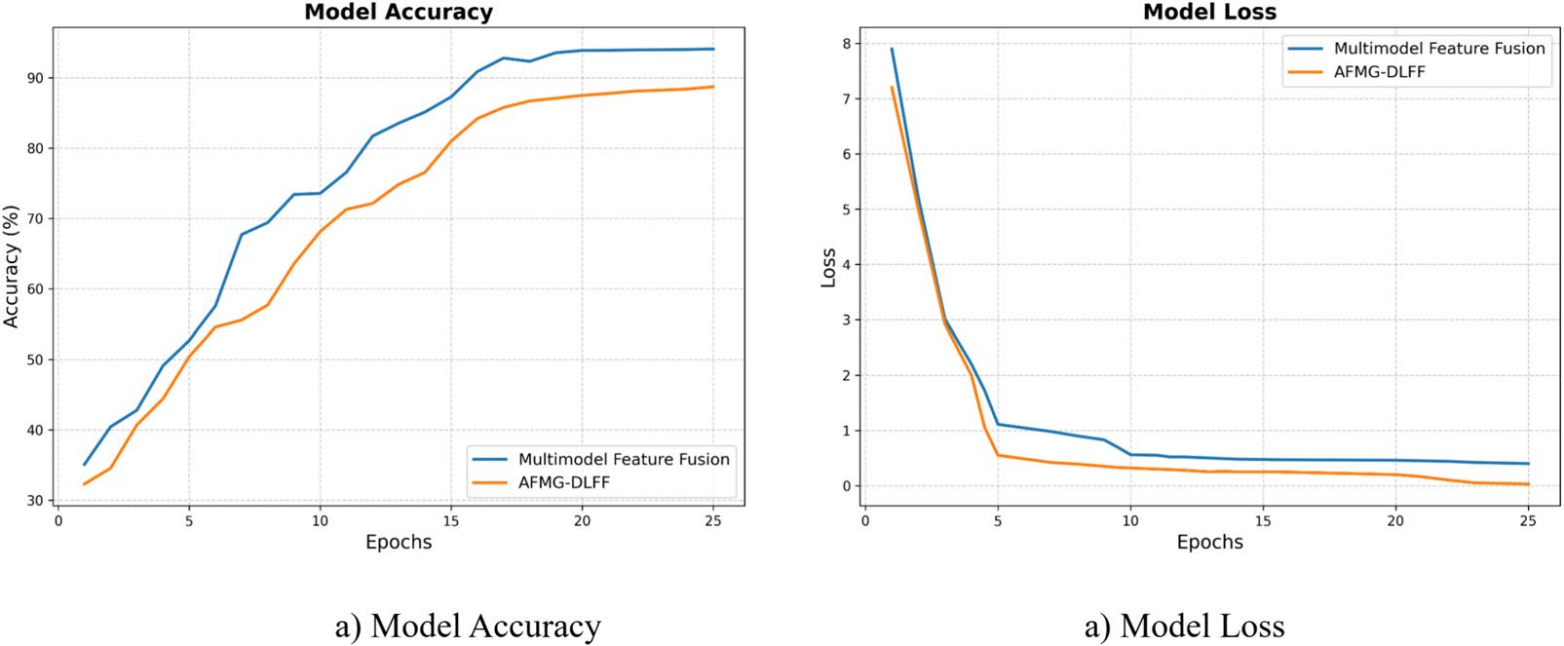
(**a**) Model Accuracy (MA) and (**b**) Model Loss (ML) curves of the proposed AFMG-DLFF approach illustrating training progression, convergence behaviour, and overall model stability across epochs.

Figure 7a, b visually reinforce these findings. The post-optimization curves demonstrate faster convergence, higher final accuracy, and smoother loss behaviour, confirming improved generalisation. Figure 7a presents the accuracy curves of the MFF and AFMG-DLFF models, where the optimised framework shows a steeper and more stable rise in accuracy. Figure 7b displays the training- and validation-loss curves, revealing a monotonic decline with minimal oscillation, indicating that GSO effectively stabilised learning and mitigated overfitting. To further counter minor overfitting observed during training, early stopping with a slightly elevated learning rate was implemented. This approach enabled rapid convergence of loss values while preventing unnecessary epochs, ensuring optimal model generalisation.

### Evaluating the results against state-of-the-art approaches

The proposed AFMG-DLFF model, which integrates both visual (extrinsic) and biochemical (intrinsic) features, was compared with selected literature-reported fruit ripeness grading approaches (Table 8). By combining these complementary modalities, the framework captures both surface-level visual cues and internal biochemical indicators, which is particularly relevant for visually uniform NDMST mangoes. The comparison indicates that AFMG-DLFF is competitive with related published methods while offering the specific advantage of multimodal extrinsic–intrinsic fusion. However, because the studies listed in Table 8 were developed on different fruit types, datasets, and experimental conditions, these comparisons should be interpreted as contextual literature comparisons rather than as direct same-dataset benchmarking across classifier families.

The *97.86%* accuracy achieved by AFMG-DLFF underscores the potential benefit of combining external and internal information for NDMST maturity grading. For cultivars with weak or visually uniform ripening cues, multimodal integration may provide a more informative representation than reliance on a single source of information alone. In this context, methods based solely on external imaging may be less suitable for varieties with limited visual discrimination, while internal sensing approaches such as NIR spectroscopy and hyperspectral imaging often require expensive specialised equipment and substantial computational resources. By integrating accessible RGB imaging with biochemical traits, AFMG-DLFF represents a practical and cost-effective strategy for this specific cultivar and experimental setting.

**Table 8:** Comparison results of grading ripeness with state-of-the-art methods.

Model	Fruit type	Analysis	Accuracy
AFMG-DLFF model	NDMST	External + Internal	97.86%
FANN^24^	NDMST	Internal	89.60%
AFGL-MC^33^	Fuji apple	External	95.81%
NIR Spectroscopy^34^	Durian	Internal	93.77%
Hyperspectral Imaging^35^	Tomato	Internal	87.52%
SqueezeNet model^36^	Mango	External	96.37%

## Conclusion

Assessing fruit maturity in cultivars such as NDMST mangoes remains challenging due to their weak external ripening cues. This study introduced AFMG-DLFF, a multimodal deep learning framework that jointly leverages RGB visual features and intrinsic biochemical traits for non-destructive maturity classification. By integrating lightweight visual encoders (DenseNet201, Inception–ResNetV2, EfficientNetV2), an FFCNN-based biochemical feature extractor, and GSO-driven hyperparameter optimisation, the system achieved a classification accuracy of 97.86%. Although GSO proved effective within the present framework, alternative hyperparameter optimization strategies were not systematically compared in this study and should be evaluated in future work. The current evaluation focused on comparisons within the proposed deep-learning framework on the same dataset; broader same-dataset benchmarking against conventional classifiers was outside the scope of the present study and should be explored in future work. The fusion of RGB and biochemical cues demonstrates that high predictive performance can be achieved without the need for costly hyperspectral or thermal imaging systems, making the method practical for large-scale agricultural deployment.

To address limited and imbalanced training data, the study employed CGAN-generated images and SMOTE-balanced biochemical vectors. Because synthetic samples lack fruit-level correspondence, a class-wise pairing strategy was used, modelling the joint distribution as $\hat{p}(I,x\mid y)\approx \hat{p}(I\mid y)\hat{p}(x\mid y)$. This simplification stabilised multimodal learning while ensuring that all validation and test evaluations relied exclusively on real image–biochemical pairs. The strong generalisation performance suggests that the model captures genuine ripening-related cues rather than artefacts of synthetic augmentation, though the conditional-independence assumption may limit the modelling of finer physiological interactions.

As a proof-of-concept built from a single orchard, single harvest season, and controlled acquisition setting, the present work does not yet establish robustness across broader environmental and data-source variability. In particular, season-wise and weather-related factors such as temperature, humidity, rainfall, and orchard microclimate were not explicitly incorporated into the present modelling framework, and the proposed method was not evaluated on datasets collected from different orchards, locations, seasons, or imaging conditions. Accordingly, the broader generalizability of the current results should be interpreted with caution. Future studies will expand data collection across multiple seasons and locations, incorporate weather-linked metadata where available, evaluate the framework on independent external test sets, and assess robustness under varying acquisition conditions to more rigorously establish cross-source and environmental applicability. Larger multi-season datasets will also enable controlled ablation studies without synthetic augmentation to validate the isolated contribution of CGAN and SMOTE. Reducing dependence on labelled biochemical data will be pursued through self-supervised and semi-supervised multimodal learning. The framework’s modularity further supports extension to other fruits such as papaya, tomato, and apple, as well as future deployment on edge devices, IoT platforms, and drone systems for real-time maturity assessment.

Overall, AFMG-DLFF demonstrates that combining accessible RGB imaging with intrinsic biochemical cues provides a cost-effective and accurate pathway for automated fruit maturity grading, addressing the limitations of both traditional visual inspection and high-cost sensing technologies.

## Data Availability

The datasets used and/or analysed during the current study available from the corresponding author on reasonable request.
